# List of Reviewers 2022

**DOI:** 10.1192/bjo.2023.10

**Published:** 2023-08-25

On behalf of the editorial board, the Editor-in-Chief would like to thank the following for their contribution as peer reviewers in the period from January 2022 – December 2022. Their anonymous work for the journal forms the foundation to its success, and is highly appreciated.

- Amanuel Abajobir
- Melanie Abas
- Catherine Abbo
- Karim Abdel Aziz
- Anne Abe
- Riadh Abed
- Marios Adamou
- Simon Adamson
- Bruno Agustini
- Nilu Ahmed
- Olesya Ajnakina
- Oluyemi Akanni
- Qais Alemi
- Regi Alexander
- Janice Allister
- Gokay Alpak
- Najim Alshahrani
- Mohammed Al-Uzri
- Nicholas Ambler
- Kelly Anderson
- Kirstie Anderson
- Humma Andleeb
- Gerard Anmella
- James Christopher (Jim) Anthony
- Rebecca Anthopolos
- Paul Appelbaum
- Alice Arcury-Quandt
- Angeliki Argyriou
- Kevin Ariyo
- Danilo Arnone
- Teresa Arora
- Bill Arroyo
- Nargis Asad
- Nobutaka Ayani
- Oyedeji Ayonrinde
- Enrique Baca-Garcia
- Julia Bailey
- Harinder Bains
- Alison Baird
- David Baldwin
- Sarah Barber
- Lucy Barker
- Annarita Barone
- Debasish Basu
- Howard Bauchner
- Joy Noel Baumgartner
- Sallie Baxendale
- Ben Beaglehole
- Klaus Martin Beckmann
- Victoria Bein
- Caroline Bell
- Veerle Bergink
- Tomi Bergström
- Steve Berkowitz
- Marco Bertelli
- Rahul Bhattacharya
- Vishal Bhavsar
- Kamaldeep Bhui
- Renzo Bianchi
- Eleonora Bielawska-Batorowicz
- Irene Bighelli
- Jo Billings
- Marie Bismark
- Mary Bitta
- Istvan Bitter
- Graham Blackman
- Simon Blackwell
- Mariia Bocharova
- Rebecca Bogaers
- Lana Bojanić
- Jessica Bone
- Hans Bosma
- Muriel Boucart
- Juliette Bouzy
- Mhairi Bowe
- Phillip Boyce
- Jane Boydell
- Owen Bradfield
- Elspeth Bradley
- Rachel M. Brand
- Eva Janina Brandl
- Philip Braude
- Beatrice Bravi
- Augusto Brenner
- Carlinde Broeks
- Lisa Brophy
- Denver M. Y. Brown
- Konstantin Brückmann
- Ronny Bruffaerts
- Maja Bruhn
- Dejan Budimirovic
- Oscar Bukstein
- Katherine Burdick
- Chris Burton
- Michael Bushey
- Ingrid Butterfield
- Haewon Byeon
- Sarah Byford
- Enda Byrne
- Kristin Cadenhead
- Jose Antonio Camacho Conde
- Andres Camilo Cardozo Alarcon
- Marina Carpena
- Livia Carvalho
- Cecilia Casetta
- Frederick Caston
- Caterina Ceccarelli
- Jessica P. Cerdena
- Jorge Cervilla
- Rakesh Chadda
- Kenneth Chambaere
- Amy Chandler
- Runsen Chen
- Wai Chen
- Ying-Yeh Chen
- Yun-Ling Chen
- Clare Cheng
- Edward Chesney
- Natasha Chilman
- Francesco Chirico
- Mian-Yoon Chong
- Charilaos Chourpiliadis
- Lydia Chwastiak
- David Clark
- Lisa Clarke
- Seonaid Cleare
- Caroline Clements
- Pollyanna Cohen
- Dan Cohen
- Jeremy Coid
- Rory Conn
- Yeates Conwell
- Collin Cortie
- Ken Courtenay
- Kenneth Cox
- David Coynel
- Nicolas Crossley
- George Crowther
- Mario Cruz
- Wiesław Cubała
- Kathryn R. Cullen
- Jackie Curtis
- Rachael Cvejic
- Louise Czosnek
- Elias Dakwar
- Oliver Dale
- Michael Daly
- Frances Dark
- Neil Davies
- Simon Davies
- Yogita Dawda
- Katie Dawson
- Enya Daynes
- Chiara De Panfilis
- Ian Deary
- Kamala Deka
- Jaime Delgadillo
- Defaru Desalegn
- Pushpal Desarkar
- Brecht Devleesschauwer
- Marie Di Blasi
- Annabella Digiorgio
- Lora Dimitrova
- Alyson Dodd
- Anne Doherty
- Geert Dom
- Xinwen Dong
- Martin Dorahy
- Alex Dregan
- Jack Drescher
- Konstantinos Drosos
- Coralie Dumoulin
- Dominic Dwyer
- Daniel Dyball
- Julian Eaton
- Kate Eggleston
- Anam Elahi
- Javier Escobar
- Regina Espinosa López
- Randall Espinoza
- Tatjana Ewais
- Anne Eyre
- Kathleen Falster
- Chao Fang
- Nicolas Farina
- I. Feldman
- Matthäus Fellinger
- Haihua Feng
- Laura Ferraro
- Anne Finucane
- Claudia Fischer
- Helen Fisher
- Cecilie Fitzgerald
- Maèva Flayelle
- Lena Flyckt
- Cheryl Foo
- Tamsin Ford
- Andrew Forrester
- Luciana Forte
- Maria Florencia Forte
- James Foulds
- Jay Fournier
- Tanja Franciskovic
- Sophia Frangou
- Paul French
- Bridget Frese
- Louise Frisen
- Thomas Frodl
- Yu Fu
- Romayne Gadelrab
- Rafael Gafoor
- Alexandra Gaillard
- Hanga Galfalvy
- Peter Gallagher
- Satheesh Gangadharan
- Satheesh Kumar Gangadharan
- Maria Gardani
- Elena Garralda
- Fiona Gaughran
- Graham Gee
- Siobhan Gee
- Gabriela Gehlen
- Fulei Geng
- Raluca Georgescu
- Deepak Ghadigaonkar
- Kristina Gicas
- Neeraj S Gill
- Lucas Giner
- Daniel Gittins Stone
- Paul Glue
- Sundar Gnanavel
- Chinar Goel
- Tomasz Gondek
- Sarah Goodday
- Christopher Gordon
- David Graham
- Matthew Grainge
- Brad Grant
- Karen Gregg
- Sian Griffiths
- Molly Gromatsky
- Raz Gross
- Denford Gudyanga
- Izabela Guimarães Barbosa
- David Gunnell
- Devika Gupta
- Mayank Gupta
- Ian Gutierrez
- Tomas Hajek
- Kyu-Man Han
- Mark Hanly
- Joyce Harrison
- Alex Haslam
- Tanya Hauck
- Christopher Haxton
- Caroline Hayes
- Katie Hazelgrove
- Qinghua He
- Timea Helter
- Jeisson Hernandez
- Morten Hesse
- Paul Hicks
- Julie Highfield
- Alannah Hillmer
- Roger CM Ho
- Frank Holloway
- Dawn Holmes
- Philipp Homan
- Xia Hong
- Matthew Hoptman
- Malcolm Hopwood
- Christoph Hörmann
- Matthew Hotopf
- Louise Howard
- Shaohua Hu
- Dieneke Hubbeling
- Vyv Huddy
- Jon Hunter
- Sharon Hunter
- Zoe Hunter
- Sarah Huque
- Ji-Won Hur
- Mark Huthwaite
- Eduardo Iacoponi
- Momoka Igarashi
- George Ikkos
- Maree Inder
- Jeremy Isaacs
- Farhadul Islam
- Sonia Israel
- Adela Isvoranu
- Iliyan Ivanov
- Jun Iwatani
- Srividya Iyer
- Pamela Jacobsen
- Glorianna Jagfeld
- Staffan Janson
- Sameer Jauhar
- Syed Javaid
- Eranda Jayawickreme
- Oskar Jefsen
- Luke Jelen
- Jessica Jones Nielsen
- Brett Jones
- Edgar Jones
- Stephen Jones
- Jenny Jordan
- Mark Jordans
- Anders Jorgensen
- Martin Jorgensen
- Mario Juruena
- Michael Kaess
- Ashraf Kagee
- Alexander Kaltenboeck
- Richard Kanaan
- Hitoshi Kaneko
- Symon Kariuki
- Takahiro Kato
- Tamar Katz
- Kenneth Kaufman
- Harneet Kaur
- Brian Keane
- James Kelly
- Christopher Kemp
- Philip Kemp
- Michael Kerr
- Mike Kerr
- Jess Kerr-Gaffney
- Nasar Khan
- Kaveh Khoshnood
- N. Khoury
- Sarah Khwaja
- Helen Killaspy
- Seung Hoon Kim
- Deborah Kinnear
- Patricia Kinser
- Tayyeba Kiran
- James Kirkbride
- Steve Kisely
- Anke Kobach
- Shinsuke Koike
- Venkat Kolli
- E. Kolshus
- Emma Kopra
- Dan Koren
- Michał Kosowski
- Christos Kouimtsidis
- George Koulierakis
- Ann-Katrin Kraeuter
- Annegret Krause-Utz
- Christa Kruger
- Karolina Krysinska
- Melike Kucukkarapinar
- Daniel Kuehnle
- Jiro Kurata
- Shin Kurose
- Kristie Ladergard
- Johanna Lake
- Séverine Lannoy
- Matthew Large
- Rachel Latham
- Richard Laugharne
- Naomi Launders
- Stephen Lawrie
- Lauren Lebois
- Allen TC Lee
- Christopher Lee
- Eunjin Lee
- Rachel Lee
- Sze Chim Lee
- Wanhyung Lee
- William Lee
- Sophie Legge
- Lonneke Lenferink
- Aiste Lengvenyte
- Eric Lenze
- Pablo León-Ortiz
- Frederick Lepore
- Peter Lepping
- Stefan Leucht
- Alexander Ryan Levesque
- Roberto Lewis-Fernandez
- Yan Leykin
- Cheng-Ta Li
- Jialiang Li
- Yanhui Liao
- Paul Lichtenstein
- Haiqun Lin
- Ping-I Lin
- Jean-Pierre Lindenmayer
- Valeria Lino
- Jennie Lister
- Dongjing Liu
- Shi-Kai Liu
- Xiaoqin Liu
- Pablo Lopez
- Anikó Lovik
- Tania Lucci
- Mario Luciano
- Farah Lunat
- Yona Lunsky
- Danielle Lutgens
- Alexandra Lysova
- Naoise Mac Giollabhui
- Waleska Regina Machado Araujo
- Sheri Madigan
- Paola Magioncalda
- Gin Malhi
- Pedro Manfro
- John Mann
- Victoria Manning
- Sarah Markham
- Niina Markkula
- Joanna Maselko
- Julian Mason
- Sharna Mathieu
- Rosalind McAlpine
- Susan McGurk
- Sally McManus
- Nick Meader
- Adrian Meehan
- Anna Meijer
- Onno C. Meijer
- Habtamu Mekonnen
- Carlos Eduardo Menezes Amaral
- Nicholas Meyer
- Emma Millard
- Stephen Miller
- Helen Minnis
- Supriya Misra
- Kirstin Mitchell
- Francis Mitrou
- Muhammad Najib Mohamad Alwi
- Alessio Maria Monteleone
- Fhionna Moore
- Michael Mooutoussis
- Benito Morentin
- Michelle Morgan
- Elias Mpofu
- Armida Mucci
- Monika Mueller
- Marlene Mühlmann
- Marco Mula
- Roger Mulder
- David Mumford
- Esther Murray
- Ora Nakash
- Prathiba Natesan Batley
- Laura Nawijn
- David Ndetei
- Adrian Neal
- Ahmed Negida
- Maria Antoinetta Nettis
- Angela Nickerson
- Maria Niemi
- Jeanet Nieuwenhuis
- Stevan Nikolin
- Zlatko Nikoloski
- Joanne Noblett
- Rhedeya Nury Nodi
- Evangelos Ntontis
- Abraham Nunes
- John-Jose Nunez
- Aileen O′Brien
- Claire M. C. O′Connor
- Graeme O′Connor
- Rory O′Connor
- David Okai
- Sam Okpaku
- Junko Okuyama
- Paula Oliveira
- Dominic Oliver
- Kristine L. Rae Olmsted
- Conor OLuanaigh
- Holly O′Rourke
- Francisco Ortega
- Mugtaba Osman
- Michael Ostacher
- Erika Ota
- Simple Ouma
- Femi Oyebode
- Andrea Panzavolta
- Carmine Pariante
- Gordon Parker
- Jeniffer Parker
- Peter Parry
- Kerstin Paschke
- Jyotishman Pathak
- Tess Patterson
- Julia Pei
- Karl Peltzer
- Robert B. Penfold
- Francesca Penner
- Anssi Perakyla
- Manuel Pereira Herrera
- David Perez
- Florence Perquier
- Amanda Perry
- Eva Petkova
- Emily Petti
- Theodore Petti
- James Phelps
- Ursula Philpot
- Peter Phiri
- Vanessa Pinfold
- Mariana Pinto Da Costa
- Nathan Pipitone
- Alexandra Pitman
- Oleguer Plana-Ripoll
- Michael Pluess
- Rob Poole
- David Popovic
- Bryony Porter
- Richard Porter
- Jonathan Posner
- Homa Pourriyahi
- Heidi Preis
- Annabel Price
- Eleonora Prina
- Tareq Quassem
- Yann Quide
- Leah Quinlivan
- Samah Rabei
- Kutlwano Ramaboa
- Rebecca Randles
- Charlene Rapsey
- Aswin Ratheesh
- Jane Ravenscroft
- Cheryl Rees
- Claud Regnard
- Lennart Reifels
- Joe Reilly
- Thomas Reilly
- Antje Reinders
- David Ring
- Gail Robinson
- Jo Robinson
- Louise Robinson
- Paul Robinson
- Humbelina Robles Ortega
- Gabriel Robles
- Natalia Pessoa Rocha
- Modesto Rolim Neto
- Stuart Rosen
- Joshua Rosenblat
- Chayim Rosensweig
- Sarah Rowe
- Ashok Roy
- Paola Rucci
- Chianchong Ruengorn
- Jorun Rugkasa
- Christopher Ryan
- Wonjung Ryu
- Martha Sajatovic
- Naomi Salisbury
- Tatiana Salisbury
- Jay Salpekar
- Zainab Samaan
- Lamiya Samad
- Musa Sami
- Elizabeth Sampson
- Clara Sancho-Domingo
- Grant Sara
- Amir Sariaslan
- Norman Sartorius
- Salvatore Sarubbi
- Rob Saunders
- Kapil Sayal
- Steven Schachter
- Yolanda Schlumpf
- Robert Schoevers
- Johannes Schroeder
- Carlo Schuengel
- Peter Schulte
- Jan Scott
- Maria Seidel
- Edward Selby
- Lucas Sempe
- Tricia Seow
- Gianluca Serafini
- Jerrie Serrell
- Vaheshta Sethna
- Pak Chung Sham
- Rohit Shankar
- Jalil-Ahmad Sharif
- Lisa Sharwood
- Richard Shaw
- Rory Sheehan
- Mark Shevlin
- Wendy Shoesmith
- Amit Shrira
- Tian-Mei Si
- Steven Silverstein
- Fiona Sim
- Almog Simchon
- Gregory Simon
- Judit Simon
- Emiliana Simon-Thomas
- Mirjam Simoons
- Alexander Simpson
- Daisy Singla
- Dan Siskind
- Nikolaos Smyrnis
- Daya Somasundaram
- Hye-Gyeong Son
- Emma Soneson
- Marion Spijkerman
- Elizabeth Spitzer
- Preeyaporn Srasuebkul
- Paul St John-Smith
- Helle Stangeland
- Robert Stanton
- Thomas Steare
- Rachel Steele
- Simona Ștefan
- Klaas Enno Stephan
- Ruth Stephen
- Andrew Steptoe
- Harriet Stewart
- Robert Stewart
- Gorazd Stokin
- Rona Strawbridge
- Rebecca Strawbridge
- Juriaan Strous
- Aikaterini Strouza
- Chia-Ting Su
- Yi Nam Suen
- Ruqayya Sulaiman-Hill
- Shihua Sun
- Lois Surgenor
- Georgina Sutherland
- Zoe Swithenbank
- Ying-Hsuan Tai
- Akihiro Takamiya
- Nori Takei
- Sandila Tanveer
- Tim Tanzer
- Laila Tata
- Alvin Kuowei Tay
- Rosie Taylor
- Devin Terhune
- Jagadisha Thirthalli
- Lindsay Thomson
- Elena Toffol
- Samuel Tomczyk
- Yongsheng Tong
- John Torous
- Derek Tracy
- Ulrich Tran
- Robert Trestman
- Samuel Tromans
- Konstantinos Tsamakis
- Hector Tsang
- Angeliki Tsapara
- Dimosthenis Tsapekos
- Stuart Turner
- Natasha Tyler
- Michael Udedi
- Rachel Upthegrove
- Patrizia Velotti
- Pieter Ventevogel
- Lucia Verginer
- Manuel Villareal
- Louiza Voniati
- Fredrik Walby
- Jo Waller
- Malin Wallin
- Zach Walsh
- Martin Walter
- Yuhui Wan
- Angel Wang
- Liang-Jen Wang
- Danuta Wasserman
- Marielle Wathelet
- Deborah Wearne
- Sally Weinstein
- Myrna Weissman
- Charlie Welch
- Perla Werner
- James West
- Karen Wetherall
- Kath Wilkinson
- Suzanne Willey
- Chris Williams
- Claire Williams
- Richard Williams
- Claire Wilson
- Toby Wise
- Dumindu Witharana
- Emma Wolverson
- Ben Hoi-Ching Wong
- Amanda Woodrow
- George E. Woody
- Adam Wrzeciono
- Chi-Shin Wu
- Jingwei Wu
- Fan Yang
- Min Yang
- Philip Yanos
- Zeng Jie Ye
- Naohiro Yonemoto
- Kazunari Yoshida
- Allan Young
- Yu Yu
- Kai Yuan
- Sedigheh Zabihi
- Roland Zahn
- Charles Zeanah
- Scott Zeller
- Kai Zhang
- Nanhua Zhang
- Shuo Zhang
- Shannon Zhong
- Janneke Zinkstok
- Hannah Ziobrowski

